# Rational design of a protein that binds integrin α_v_β_3_ outside the ligand binding site

**DOI:** 10.1038/ncomms11675

**Published:** 2016-05-31

**Authors:** Ravi Chakra Turaga, Lu Yin, Jenny J. Yang, Hsiauwei Lee, Ivaylo Ivanov, Chunli Yan, Hua Yang, Hans E. Grossniklaus, Siming Wang, Cheng Ma, Li Sun, Zhi-Ren Liu

**Affiliations:** 1 Department of Biology, Georgia State University, Atlanta, Georgia 30303, USA; 2 Department of Chemistry, Georgia State University, Atlanta, Georgia 30303, USA; 3 Department of Ophthalmology, Eye Center, Emory University, Atlanta, Georgia 30322, USA; 4 Center for Diagnostics and Therapeutics, Amoytop Biotech Inc., Xiamen 361028, China

## Abstract

Integrin α_v_β_3_ expression is altered in various diseases and has been proposed as a drug target. Here we use a rational design approach to develop a therapeutic protein, which we call ProAgio, that binds to integrin α_v_β_3_ outside the classical ligand-binding site. We show ProAgio induces apoptosis of integrin α_v_β_3_-expressing cells by recruiting and activating caspase 8 to the cytoplasmic domain of integrin α_v_β_3_. ProAgio also has anti-angiogenic activity and strongly inhibits growth of tumour xenografts, but does not affect the established vasculature. Toxicity analyses demonstrate that ProAgio is not toxic to mice. Our study reports a new integrin-targeting agent with a unique mechanism of action, and provides a template for the development of integrin-targeting therapeutics.

## Abstract (web summary)

Integrins are transmembrane proteins that have important roles in cell adhesion and signalling. Here the authors design a therapeutic protein that binds integrin α_v_β_3_, has anti-angiogenic activity, and reduces tumour growth in xenograft models, while being seemingly well tolerated.

## Graphical abstract

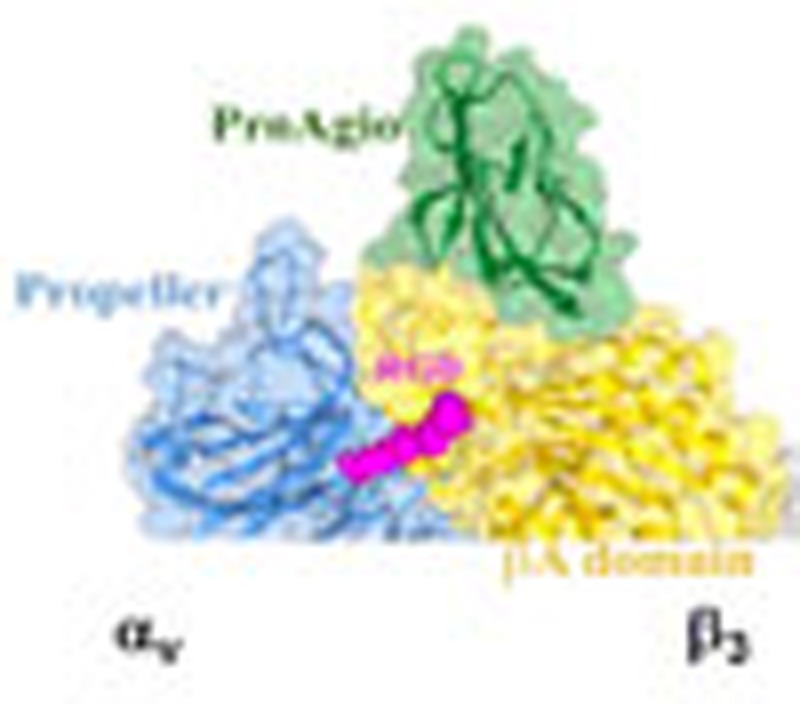

Integrins are heterodimeric (combinations of different α and β subunits) cell surface receptors. These molecules not only play critical role for the cell adhesion to extracellular matrix (ECM) but also function as an inside-out and outside-in bidirectional signalling molecules to allow cell response to many biological cues^1^,^2^. Abnormal expression of different pairs of integrins often associate with development and progression of various pathological conditions^3^,^4^,^5^,^6^. Due to unique expression patterns and functionality of integrin α_v_β_3_ in angiogenic endothelial cells, activated macrophages, metastatic cancer cells and matured bone-resorbing osteoclast cells^7^,^8^,^9^,^10^, this pair of integrins has been intensively studied as a potential target for development of anti-angiogenic and anti-inflammatory drugs^11^,^12^,^13^,^14^. Studies yield a number of successful examples. Among them are various antibodies against this integrin^15^, and most recently, Cilengitide, a Arg-Gly-Asp (RGD)-based peptidomimetic^16^,^17^. Nevertheless, most of the current approaches in development of therapeutics targeting integrin focus on ligand binding by using antibodies, cyclic peptides, disintegrin, peptidomimetics and small-molecular antagonists^15^,^18^,^19^. A major drawback of targeting ligand binding of integrin is the activation of integrin signalling by the developed agent, which largely limit the clinical success of the integrin ligand-based antagonist/agonist. There is an urgent need to develop agents that target integrin at sites other than ligand-binding site. We report here the development of a new class of therapeutic protein agent by rational protein design. The designed protein targets integrin α_v_β_3_ at a novel site, and triggers apoptosis of integrin α_v_β_3_-expressing cells via recruitment and activation of caspase 8 to the cytoplasmic domain of integrin β_3_. *In vitro* and *in vivo* experiments demonstrate that the designed protein is very effective as an anti-angiogenic agent, providing a confirmation for the specific targeting of integrin α_v_β_3_ by the designed protein agent.

## Results

### Designing a protein agent binds to a novel site of integrin α_v_β_3_

**Figure 1:**
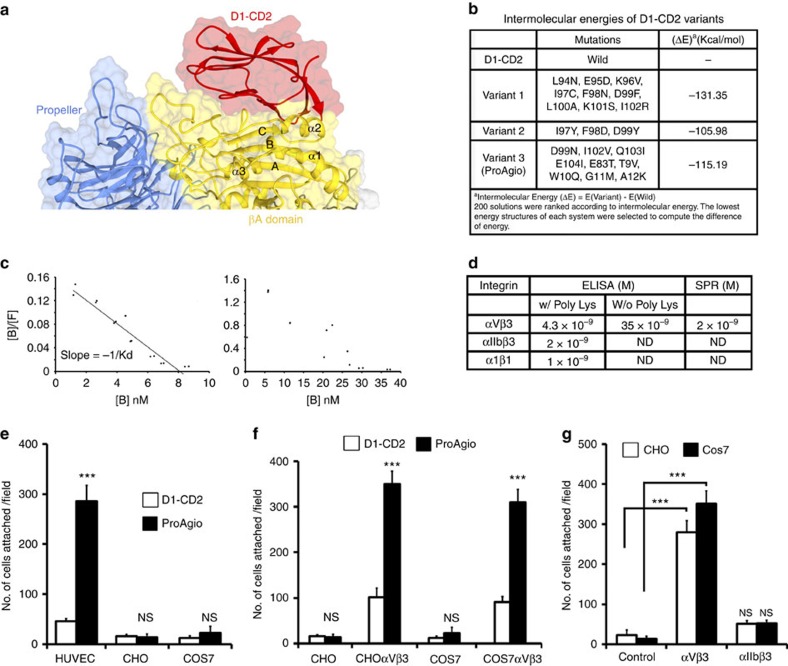
Design of protein agent ProAgio binds to integrin α_v_β_3_ at a novel site (**a**) Docking of D1-CD2 to βA domain of integrin β_3_ using software Haddock 2.1. The regions form the D1-CD2 docking site are highlighted. (**b**) Docking of D1-CD2 and three variants (mutations indicated) to βA domain of integrin β_3_ as in **a**. The (ΔE) is the difference of docking energy between D1-CD2 and a variant calculated by the docking programme. (**c**) The representative Scatchard plots of binding data of binding of ProAgio to integrins α_v_β_3_ in the presence of 3 mM polyLys (Left) and absence of polyLys (Right). The dots are binding data. Solid line in left is fitting line. The fitting line clearly indicates *n*=1 for one-to-one binding. (**d**) The deduced dissociation (Kd) constants of ProAgio to integrins α_v_β_3_, α_IIb_β_3_, and α_1_β_1_ from the ELISA and SPR binding analyses. The n.d means the Kd could not be or were not determined. (**e**,**f**) Attachment of indicated cells to culture plate on which ProAgio (filled bars) or D1-CD2 (open bars) was coated was measured by counting the cells that attached to the plate after washing. (**g**) Attachment of CHO (open bars) or COS-7 (filled bars) cells with/without (control) expression of α_v_β_3_ or α_IIb_β_3_ to culture plate on which ProAgio was coated was measured by the same cell counting as in (**e**) and (**f**). The control is CHO cells without integrin expression. The error bars in **e**,**f** and **g** are s.d. from measurement of five independent experiments. NS *P*>0.05, ***P<0.001 calculated by unpaired two-tailed Student's *t*-test. ND, not determined; NS, not significant.

We employed an approach of *in silico* and *in situ* analysis to search for proteins that potentially bind to integrin α_v_β_3_ at a site other than ligand binding site. We earlier observed a very weak affinity of domain 1 of both human and rat CD2 (referred to as D1-CD2), the proteins that were well studied in our laboratories^20^,^21^, to the integrin α_v_β_3_. Thus, we particularly attempted to dock D1-CD2 to various sites of integrin α_v_β_3_. Because of the functional importance of βA domain of β_3_ in ligand binding and integrin signalling^22^, we focused our attentions on the βA domain. To validate our docking method, we first docked a physiologic ligand of integrin α_v_β_3_, the tenth type III RGD domain of wild-type fibronectin to integrin α_v_β_3_. The RGD domain docking completely matched the crystal structure of the complex by Van Agthoven*et al*.^23^ (Supplementary Fig. 1a), indicating our docking method is appropriate. With the validation of our docking method, we carried out a randomized docking to the exposed surfaces of βA domain, D1-CD2 docked to a novel site, a groove in βA domain of β_3_ formed by the ‘α2 helix', ‘B-C loop' and ‘α2–α3 loop' (referred to as βA-groove), with various different orientations (Fig. 1a, Supplementary Fig. 1b). However, due to lack of strong contacts with residues of the βA-groove, the docking did not provide a favourable energy. To improve the docking, we made several mutations on D1-CD2 residues that may form important interactions with βA domain. Additional mutations were also introduced to counter potential structure disturbance and to maintain stable β-sheet packing. As expected, the docking-energies of these D1-CD2 variants on the βA-groove were substantially decreased (Fig. 1b). To verify the docking of D1-CD2 variants to integrin α_v_β_3_, we re-performed the docking of variant 3 with another available crystal structure of integrin α_v_β_3_ (4MMZ)^23^. The variant was docked to the same site of βA-groove. Due to structure difference between 1L5G and 4MMZ, the variant twisted about 10–15 degree in orientation in docking to 4MMZ (Supplementary Fig. 1c).

To test the results of computational modelling/docking, the rationally designed D1-CD2 variants were expressed in bacterial *Escherichia coli* and subsequently purified. Due to solubility, stability and other parameters, we chose one variant (variant 3 in Fig. 1b, which we call ProAgio) to carry out extensive characterizations. ProAgio exhibited structural properties very similar to that of the parental protein as demonstrated by the ^1^H-NMR (Supplementary Fig. 1d), far ultraviolet CD, and fluorescent spectra analyses, indicating that the engineered protein was well folded. We carried out binding analyses to determine the binding affinity and stoichiometry of ProAgio and integrin α_v_β_3_ interaction. We first performed ELISA-based binding assays. Scatchard plot of the binding data indicated that the ProAgio and integrin α_v_β_3_ binding could not fit into a one-to-one binding mode (Fig. 1c). However, in the presence of 3 mM of polyLys, the ProAgio and integrin α_v_β_3_ binding fitted well into a one-to-one binding mode with a deduced dissociation constant (Kd) of 4.3 nM (Fig. 1c,d). The results suggest that ProAgio may interact with integrin α_v_β_3_ by both specific and non-specific interactions, and the non-specific interaction is most likely due to protein surface charges. To test whether ProAgio and integrin α_v_β_3_ interaction is α_v_β_3_ specific, the ELISA-based binding analyses were also performed with other two pairs of integrin. Clearly, ProAgio interacted weakly with other two integrin pairs in the presence of polyLysine (Fig. 1d). To verify the ELISA-based binding analyses, we also carried out surface plasmon resonance (SPR)-binding studies. To avoid the problem of non-specific interactions, SPR binding experiments were carried out using PEGylated ProAgio (30 kDa PEG chain). PEGylated ProAgio bound to integrin α_v_β_3_ via an one-to-one binding mode with an affinity of deduced Kd 2 nM (Supplementary Fig. 1e and Fig. 1d), consistent with the ELISA-based binding analyses. The ProAgio and integrin interaction was metal ion (Ca^2+^) dependent, as addition of EGTA abrogated the interaction, indicating that maintenance of local structure of the βA domain is critical for the interaction.

To further verify the ProAgio and integrin α_v_β_3_ interaction, we carried out cell attachment assays using culture plate coated with ProAgio. HUVEC cells have very high levels of α_v_β_3_ expression (Supplementary Fig. 2a). The cells attached strongly to the ProAgio-coated plates (Fig. 1e). CHO and COS-7 cells do not express α_v_β_3_ (refs 24, 25). The cells did not attach to the plates. We then exogenously expressed α_v_β_3_ in CHO and COS-7 cells (Supplementary Fig. 2b) and performed the same attachment analyses. Evidently, the α_v_β_3_-expressing CHO and COS-7 cells attached to the ProAgio-coated plates (Fig. 1f). As a control, CHO and COS-7 cells expressing integrin α_IIb_β_3_ did not attach to the plate (Fig. 1g). Integrin α_v_ can authentically pair with β_3_, β_5_ and β_6_. To further test the specificity of ProAgio–integrin interaction, we carried out attachment assays with COS-7 cells expressing integrin α_v_β_3_, α_v_β_5_ and α_v_β_6_ (Supplementary Fig. 2c). Clearly, only the cells expressing α_v_β_3_ attached strongly to the plate, while α_v_β_5_-expressing cells exhibited a weak attachment (over threefold less than that of α_v_β_3_-expressing cells). The α_v_β_6_-expressing cells almost did not attach to the plate (Supplementary Fig. 2d). The attachment assays suggested that ProAgio interacted with integrin α_v_β_3_ on the cell surface. Interaction of ProAgio with the integrin was further verified by co-immunoprecipitation of ProAgio with integrin β_3_ in HUVEC cell extracts (Supplementary Fig. 2e).

**Figure 2:**
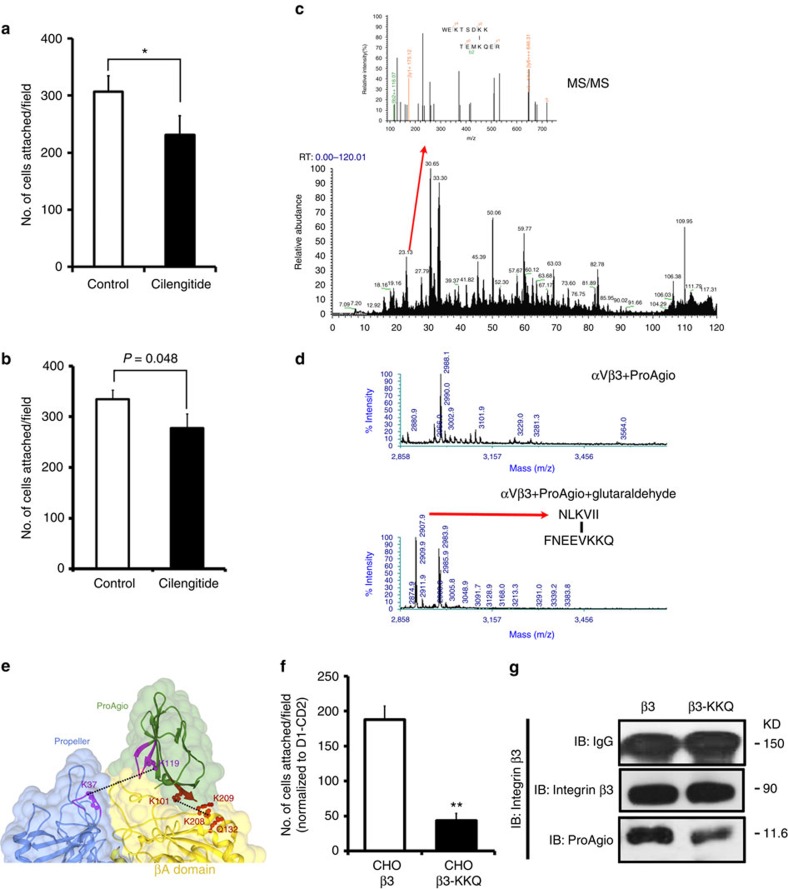
ProAgio binds to integrin α_v_β_3_ at the designed site. (**a**,**b**) Attachment of HUVEC cells to ProAgio coated plates. The attachment assays were carried out in the presence of 5 μM of Cilengitide or BSA as control (**a**), or 40 μg ml^−1^ antibody LM609 or IgG as control (**b**). (**c**) The MS spectrum of peptide fragments resultant from trypsin digestion of crosslinks of ProAgio with integrin α_v_β_3_ using BS2G as a crosslinker. The call-out is the enlarged region MS2 (indicated by the arrow) containing ProAgio–integrin crosslinking peak and analysed by MS/MS. The sequence on top is derived crosslinking sequence from MS/MS using pLink. (**d**) The MS spectrum of peptide fragments resultant from trypsin digestion of (Bottom) crosslinks of ProAgio with integrin α_v_β_3_ using glutaraldhyde as crosslinker and (Upper) ProAgio–integrin α_v_β_3_ mix without crosslinking. The deduced sequences of the 2907.9 Da peaks from MS/MS analyses are indicated. (**e**) Docking model of ProAgio with integrin α_v_β_3_ using Haddock 2.1 program by introducing the crosslinking restrains. The crosslinks are indicated by dotted lines. The mutation sites of the integrin β3 are indicated in red. (**f**) Attachment of CHO cells with expression of integrin α_v_β_3_ wild type or the mutant (β3-KKQ) to ProAgio/Vitronectin coated plates. The cell attachment to ProAgio are normalized to cell attachment to D1-CD2. (**g**) Co-immunoprecipitation of β3 or mutant (β3-KKQ) with ProAgio (IP:integrin β3) from the HUVEC cell lysate was examined by immunoblot (IB:ProAgio). The cells were treated with 5 μM ProAgio for 6 h before the extracts preparation. IB:IgG indicate the amounts antibody used in IP. Immunoblot of integrin β_3_ (IB:integrin β_3_) indicates amount of β_3_ was precipitated down in the co-IPs. Error bars in **a**,**b** and **f** are s.d. from measurement of five independent experiments. *P>0.05, **P<0.005 calculated by unpaired two-tailed Student's *t*-test.

We next analysed whether ProAgio indeed bound to the βA-groove. As predicted in our docking, ProAgio binding should not compete with RGD binding (they bind to different sites). We carried out an attachment assay, HUVEC cells were first incubated with Cilengitide, and subsequently assayed for the attachment to ProAgio in the presence of Cilengitide. Clearly, the presence of RGD did not prevent HUVEC cells from attaching to ProAgio, (Fig. 2a). Antibody LM609, a monoclonal antibody target at the ligand-binding site of integrin α_v_β_3_, is known to recognize the ligand binding site of integrin α_v_β_3_. Thus, we tested whether the presence of LM609 would block the ProAgio binding. Evidently, LM609 did not affect the interaction between ProAgio and integrin α_v_β_3_ (Fig. 2b). To further test whether ProAgio indeed binds to the βA-groove, we carried out chemical crosslink of ProAgio with purified recombinant integrin α_v_β_3_ using Bis(Sulfosuccinimidyl)-glutarate (BS2G, Supplementary Fig. 3a) as a crosslinker. Crosslinking followed by trypsin digestion and liquid chromatography-mass spectrometry (LC-MS) proteomic analyses indicated that ProAgio WEKTSDKK (aa 35–43) was crosslinked to integrin α_v_ at the TEMKQER (aa 116–122; Fig. 2c, for details see online Supplementary Materials, and Supplementary Fig. 3b,e). We did not detect crosslinks of ProAgio with integrin β_3_ using BS2G as a crosslinker. It is possible that the bulky crosslinking reaction groups of BS2G prevented ProAgio from crosslinking to integrin β_3_. To further unambiguously determine the ProAgio-binding site, we performed the crosslinking using another crosslinker glutaraldehyde (Supplementary Fig. 3a). Regions of NLKVII (aa 99–105) of ProAgio crosslinked to FNEEVKKQ (aa 203–210) of β_3_ by glutaraldehyde (Fig. 2d, for details see online Supplementary Materials, and Supplementary Fig. 3c). With the binding and crosslink results, we re-docked ProAgio to integrin α_v_β_3_ with application of the crosslinking restrains using Haddock 2.1 (Fig. 2e). The modelling revealed several important contacts between ProAgio and the integrin. Guided by the modelling, mutations were introduced into β_3_ (for example, K233A and K234A). Computations showed that the mutations weakened the ProAgio and the integrin docking (the docking energy increased by 107 kcal per mole in the same docking model as shown in Fig. 2e). The mutations indeed reduced the attachment of the mutant expressing CHO cells (Supplementary Fig. 3d) to ProAgio (Fig. 2f), and abolished ProAgio and integrin β_3_ co-immunoprecipitation (Fig. 2g). Thus, we concluded from our binding, crosslinking and mutational studies that ProAgio interacted with integrin α_v_β_3_ at the βA-groove.

**Figure 3:**
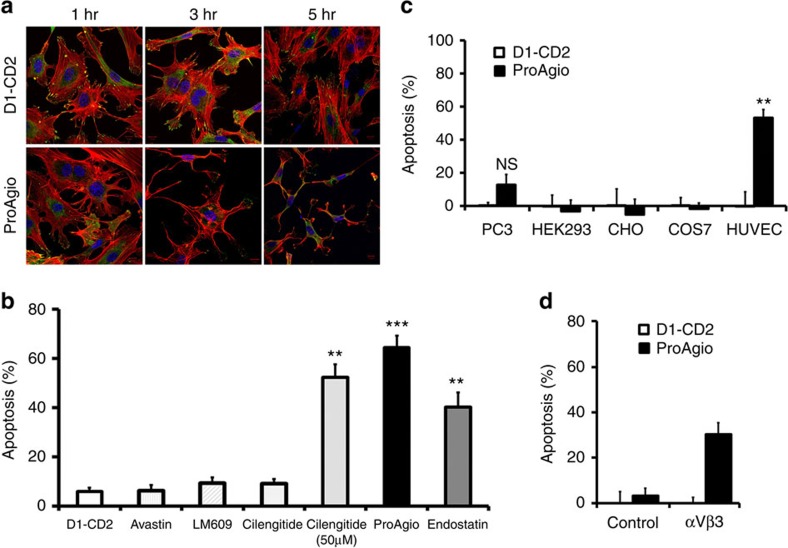
ProAgio induces apoptosis via targeting integrin α_v_β_3_. (**a**) Representative confocal microscopic images of HUVEC cells stained by Rhodamine phalloidin (red), antibody against vinculin (green) and DAPI (blue). The cells were treated by the indicated protein agents for indicated times. (**b**–**d**) Apoptosis of various different cells ((**b**) HUVEC, (**c**) indicated cells and (**d**) COS-7 with (α_v_β_3_) or without (Control) integrin α_v_β_3_ expression) was measured by cell counting 24 h after treatment with indicated agents. Cell apoptosis is presented as % apoptosis by defining the apoptosis of untreated cells as 0%. In all treatments, 5 μM (final concentration) of all agents were used except in one case in **b** wherein 50 μM Cilengitide was used (indicated). Error bars in **b**,**c** and **d** are s.d. from measurement of five independent experiments. NS *P*>0.05, **P<0.005, ***P<0.001 calculated by unpaired two-tailed Student's *t*-test. Scale bar in **a** is 10 μm. NS, not significant.

To test whether ProAgio treatment would have effects on the integrin activity, we analysed the formation of actinfilament stress fibres and the focal adhesion complex in integrin α_v_β_3_ expression HUVEC cells. Evidently, the actinfilament stress fibres decreased and eventually disappeared on the ProAgio treatment. The accumulation of vinculin at the tips of stress fibres were diminished on treatment (Fig. 3a), indicating disassociation of the focal adhesion complex. Furthermore, ProAgio treatment led to a rapid inactivation of FAK that was activated by attachment to ECM (Supplementary Fig. 4a). These results demonstrated that ProAgio abrogated the integrin functions in endothelial cells.

### ProAgio activates caspase 8 at the cytoplasmic domain of integrin β_3_

Preceding tests suggested that a rationally designed protein that targets a unique site of integrin α_v_β_3_ had been generated. We noted a strong effect of ProAgio in the induction of apoptosis of HUVEC cells while the cells were still attached in our previous assays. To our knowledge, this effect is different from that of other integrin-targeting agents. Thus, we assessed the effectiveness of ProAgio in the induction of cell apoptosis. ProAgio induced HUVEC cell apoptosis with EC_50_ around 1.4 μM (Supplementary Fig. 4b). We subsequently compared ProAgio side-by-side with several other agents that are known to have effects on HUVEC cells, such as Avastin, Endostar (an endostatin derivative approved for lung cancer treatment in China), LM609 and Cilengitide, using HUVEC cells. ProAgio was much more effective in inducing apoptosis than other agents (Fig. 3b). We further tested whether the effects of apoptosis induction were integrin α_v_β_3_ dependent. To this end, cell apoptosis was analysed with several cell lines, HUVEC, PC-3, HEK and COS-7 with/without exogenous expression of integrin α_v_β_3_, α_v_β_5_ and α_v_β_6_. HUVEC cells express high levels of α_v_β_3_. PC-3 cells express marginal levels of the integrin^26^. HEK cells express α_v_ but not β_3_ (ref. 27). COS-7 cells do not express α_v_β_3_ (ref. 25). ProAgio did not induce apoptosis with COS-7 and HEK cells. The protein induced apoptosis of HUVEC and COS-7 cells with exogenous expression of α_v_β_3_ integrin, and ProAgio did not induce apoptosis of α_v_β_5_ and α_v_β_6_-expressing COS-7 cells (Fig. 3c,d and Supplementary Fig. 4c). Moderate apoptosis was observed when PC-3 cells were treated with high concentration of ProAgio (>30 μM). The observations indicated that the effects of ProAgio in apoptosis induction were α_v_β_3_ dependent.

**Figure 4:**
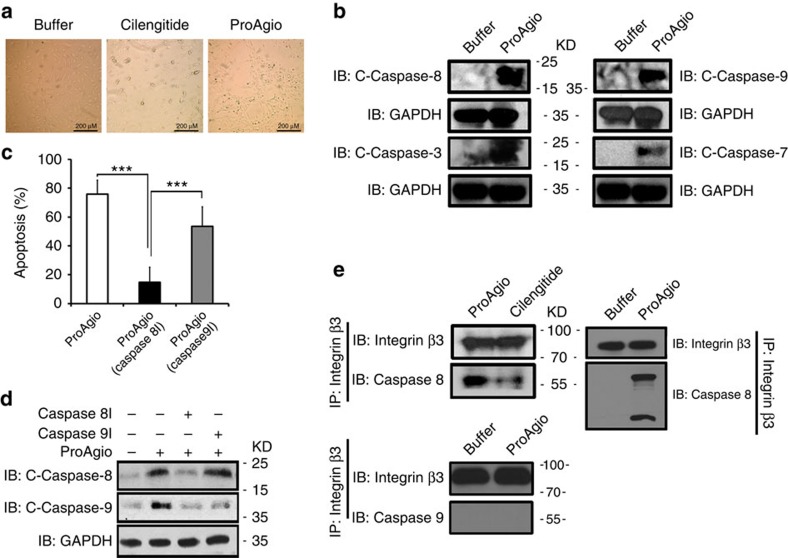
ProAgio recruits and activates caspase 8 at cytoplasmic domain of integrin β_3_. (**a**) Representative phase contrast microscopic images of endothelial HUVEC cells that were treated by indicated agents for 8 h. (**b**) Levels of indicated cleaved caspases (C-caspases) in HUVEC cell lysates were analysed by immunoblot using antibodies against various cleaved caspases. The cells were treated by 5 μM ProAgio for 10 h. (**c**) Apoptosis of HUVEC cells that were treated by 5 μM ProAgio in the presence of caspase 8/9 inhibitors was analysed by apoptosis kit. Apoptosis was presented as % Apoptosis by defining the cells that were untreated as 0%. Error bars are standard deviations from measurement of five independent experiments. (**d**) Levels of cleaved casapases 8/9 (C-caspase 8/9) in HUVEC cell lysates were analysed by immunoblot using antibody against cleaved caspase 8/9. The cells were treated with 5 μM ProAgio and caspase 8/9 inhibitor for 5 h. (**e**) Co-immunoprecipitations of caspase 8 (Upper) and caspase 9 (Bottom) with integrin β_3_ (IP:integrin β_3_) were analysed by immunoblots (IB:caspase 8/9). The cells were treated by the indicated agents 5 h before the preparation of extracts. Immunoblot of integrin β_3_ (IB:integrin β_3_) indicates amount of β_3_ was precipitated down in the co-IPs. Immunoblot of GAPDH (IB:GAPDH) in **b** and **d** are loading controls. ***P<0.001 calculated by unpaired two-tailed student t-test. Scale bar in **a** is 200 μm.

An intriguing question is how ProAgio induces endothelial cell apoptosis by binding to the βA-groove of the integrin β_3_. We noted in preceding apoptosis tests that, in contrast to the effects of Cilengitide and other integrin-targeting molecules, ProAgio treatment did not lead to HUVEC cell floating/detaching (Fig. 4a), indicating that ProAgio induced apoptosis by a mechanism that is different from anoikis. To understand the molecular mechanism by which ProAgio induces endothelial cell apoptosis, we analysed the activation of various caspases in HUVEC cells on ProAgio treatment. Caspase 8, caspase 9, caspase 7 and caspase 3 were strongly activated (Fig. 4b). We then tested whether the apoptosis induction by ProAgio was dependent on activation of an initiation caspase, for example, caspase 8 and/or caspase 9 using caspase 8 and caspase 9 inhibitors. Caspase 9 inhibitor did not abolish the effects of ProAgio on apoptosis induction, while caspase 8 inhibitor largely abolished the effects (Fig. 4c). On the other hand, caspase 9 inhibitor did not abrogate the activation of caspase 8, while the activation of caspase 9 was largely inhibited by caspase 8 inhibitor (Fig. 4d), suggesting that the caspase 8 activation mediated the effects of ProAgio. It was observed by Stupack *et al*.^25^ that unligated integrin β_3_ can trigger cell apoptosis by directly recruiting caspase 8 to its cytoplasmic domain by a mechanism of so-called integrin-mediated death (IMD). Thus, we reasoned whether ProAgio binding to the βA-groove could trigger/enhance a mechanism that is similar to that of IMD. We examined whether ProAgio treatment led to direct recruitment and activation of caspase 8 to the cytoplasmic domain of integrin β_3_ by co-immunoprecipitation. Clearly, caspase 8 was co-immunoprecipitated with integrin β_3_ on ProAgio treatment (Fig. 4e). Caspase 9 did not co-immunoprecipitate with integrin β_3_ on the ProAgio treatment (Fig. 4e). As a comparison, Cilengitide did not result in strong recruiting caspase 8 to β_3_ under the same conditions (Fig. 4e). The results suggest that ProAgio may induce endothelial cell apoptosis by a mechanism that is similar to that of IMD and is very different from that of other integrin antagonists.

### ProAgio effectively inhibits tumour growth and reduces tumour blood vessels

**Figure 5:**
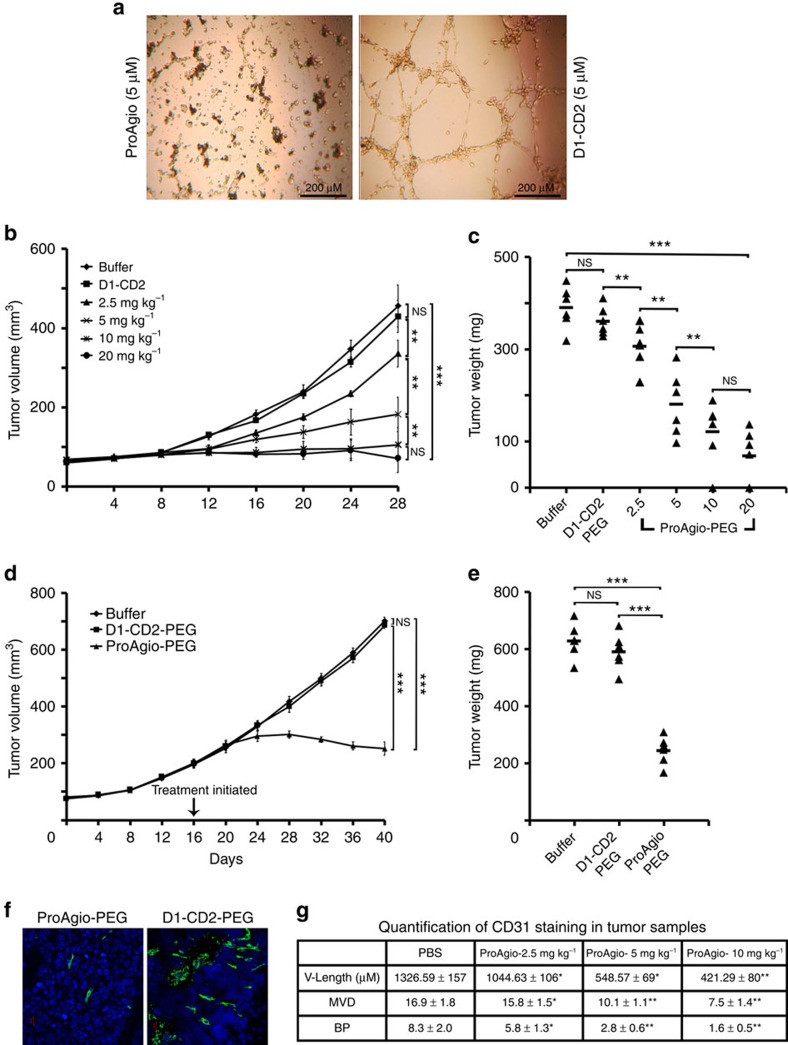
ProAgio effectively inhibits tumour growth and disrupts tumour vessels. (**a**) Representative phase contrast microscopic images of endothelial tubes of HUVEC cells under treatment of indicated agents (5 μM). (**b**–**e**) Growth of xenograft PC-3 tumours under the treatment of indicated agents was monitored by; (**b**,**d**) growth curve by measuring tumour volumes every four days, or (**c**,**e**) endpoint weights of the harvested tumours. Treatments started 5 days (**b**)/(**c**) or 17 days when tumours reached average of 250 mm^3^ in size (**d**)/(**e**) post tumour inoculations. (**f**) Representative images of immunefluorescence staining of tissue sections prepared from the harvested tumours with antibodies against mouse CD31 (Green). The blues are DAPI stains. (**g**) Quantitative analyses of vessel lengths, densities, and branch points (manually counting) of the CD31 staining of the tumour tissue sections. The vessel density and number of branch points are counted in 319.8 × 319.8 μm^2^ view field using imaging-J. The quantization was average values of randomly selected three fields in position matched 4 sections from each tumour. Error bars in **b**,**c**,**d** and **e** are s.d. from measurement of experimental six mice. NS *P*>0.05, **P<0.005, ***P<0.001 calculated by unpaired two-tailed Student's *t*-test. Scale bar in **a** is 200 μm in **f** is 20 μm. NS, not significant.

We next asked whether the integrin α_v_β_3_-targeting ProAgio would be effective in the *in vivo* experiments. We chose to test the effectiveness of ProAgio on inhibition of angiogenesis. We first examined the anti-angiogenesis activity of ProAgio by the *in vitro* endothelial tube formation assay using HUVEC cells. Evidently, ProAgio almost completely disrupted the endothelial tube (Fig. 5a). To facilitate the *in vivo* test, ProAgio was site specifically PEGylated using PEG-20 kDa, a molecule with linear-shaped PEG chain (denoted as ProAgio-PEG), at an introduced Cys residue (Supplementary Fig. 5a). The Cys mutation was introduced at a site that is away from residues involved in integrin contacts (based on computation modelling). *In vitro* tests with HUVEC cells indicated that the PEGylation did not result in significant decrease in the activity (Supplementary Fig. 5a). We test the effects of PEGylated ProAgio in angiogenesis by Matri-gel plug assays. Evidently, ProAgio inhibited angiogenesis in the Matri-gel plug assay. The activity was stronger than that of Avastin (Supplementary Fig. 5b). We further tested the effects of ProAgio in inhibition of tumour growth in animal models of cancers. We generated xenograft model of PC-3 cells. Tumour-bearing mice were intraperitoneally administered various doses of ProAgio-PEG and PEGylated host protein, and buffer saline for 20 days via one dose every other day. The treatments started at the fifth day post tumour inoculation. It was clear that ProAgio-PEG inhibited the tumour growth almost completely at the dose of 10 and 20 mg kg^−1^ (two and one tumours completely disappeared in the 20 and 10 mg kg^−1^ dose groups, respectively). As controls, the tumours grew at normal rate in mice treated with buffer and the PEGylated host proteins. The effects of ProAgio-PEG on tumour growth inhibition showed a clear dose dependency. However, the dose dependence became less significant after 10 mg kg^−1^ (Fig. 5b,c). ProAgio-PEG was also effective when administered at a later time of tumour growth (Fig. 5d,e, two tumours shrunken in size). To analyse the effects of the treatment on tumour vessels, we carried out immunostaining of CD31, an endothelial marker, with the tissue sections prepared from collected tumours. Immunostaining demonstrated that the blood vessels were markedly reduced (Fig. 5f). Quantitative analyses of the CD31 staining indicated that there were marked reductions in vessel density, branch points and vessel lengths (Fig. 5g). To further assess the effectiveness, we carried out experiments to compare side-by-side the effect of ProAgio-PEG versus Avastin and ProAgio-PEG versus Endostar using the PC-3 xenografts. ProAgio-PEG was significantly more effective in the inhibition of tumour growth compared with those of the Avastin and Endostar treatments (Supplementary Fig. 6a,b and Supplementary Fig. 6c,d). Analyses of tumour vessels showed that the ProAgio-PEG treatment led to a higher degree of vessel reduction than those of Avastin and Endostar treatment groups (Supplementary Table 1).

Tumour angiogenesis is affected by the surrounding microenvironment. Thus, we tested whether the effects of ProAgio-PEG on tumour growth and angiogenesis were also true for an orthotopic model with immune-compatible mice. We carried out similar treatment experiments with the orthotopic model of murine breast 4T-1 cells using Balb/c mice. The tumour-bearing mice were treated by same dose schedule by ProAgio-PEG or buffer as in previous treatment. The treatment started at fifth day post tumour inoculation. Evidently, there was a large difference in tumour growth between the ProAgio-PEG- and buffer-treated groups (Supplementary Fig. 6e,f), indicating that ProAgio is highly effective in inhibiting tumour growth with this orthotopic model.

### ProAgio is not toxic to mouse tissues and organs

A specific integrin α_v_β_3_-targeting agent should be less/nontoxic to existing blood vessels in normal tissue/organs. To assess whether ProAgio disrupted the non-tumour blood vessels, we prepared tissue sections from liver, lung, heart, spleen and kidney of the mice that were treated with different agents. The tissue sections were analysed by Hematoxylin staining and CD31 immunostaining. Hematoxylin staining revealed that there was no obvious abnormality in tissue anatomy structure, and no damage lesion/necrosis was observed (Supplementary Fig. 7a). Immunofluorescence staining using the anti-CD31 antibody suggested that there was no disruption or reduction of blood vessels in the liver, lung, heart and kidney on the treatments with ProAgio-PEG compared with those in the groups that were treated with the PEGylated D1-CD2 and buffer saline (Supplementary Fig. 7a). The treatments did not lead to any abnormal weight gain or loss (Supplementary Fig. 7b). To further analyse the toxicity of ProAgio, healthy CD-1 mice were intravenously administered three doses of 60 mg kg^−1^ of ProAgio-PEG in 48 h and observed for 24 days. All animals behaved normally. Blood and urine samples were collected. Examination of plasma AST, ALP, TnT, creatinine and urine albumin 48 h after the agent's administrations indicated that ProAgio-PEG at the dose did not cause damage to animal's liver, kidney and heart (Supplementary Fig. 7c).

## Discussion

We have successfully developed a protein agent targeting integrin α_v_β_3_ at a novel site by rational protein design. *In vitro* and *in vivo* experiments demonstrate the effectiveness of our designed protein. The developed agent is not toxic to non-cancerous blood vessels and other tissue/organs, providing an excellent candidate for future potential clinical development. The βA-groove is present in almost all other β-integrin. Thus, our approach may be applicable to develop agents targeting the similar βA-groove of other integrin pairs.

**Figure 6:**
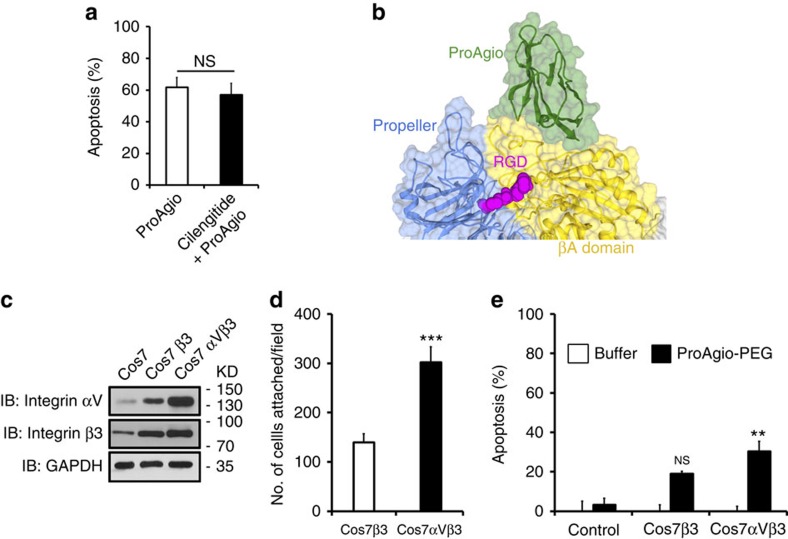
ProAgio and integrin α_v_β_3_ interaction is independent of RGD binding. (**a**) Apoptosis of HUVEC cells was measured by cell counting 24 h after treatment with 5 μM of ProAgio. The cells were incubated with 5 μM Cilengitide (filled bar) or buffer (open bar) before the ProAgio treatment. Cell apoptosis is presented as % apoptosis by defining the apoptosis of untreated cells as reference 0%. (**b**) Docking model of ProAgio (Green) with integrin α_v_β_3_ in the presence of RGD (Red) using HADDOCK 1.2 program. (**c**) Levels of α_v_ (IB: Integrin αV) and β_3_ (IB: Integrin β3) integrins in COS-7 cells with expression of β3 (COS7β3) or α_v_β_3_ (COS7αVβ3) were analysed by immunoblot of cell lysates. Immunoblot of GAPDH (IB:GAPDH) is a loading control. (**d**) Cell attachment of COS-7 cells with expression of α_v_β_3_ (COS7αVβ3) or β_3_ (COS7β3) to culture plate coated with ProAgio. The cell attachments are presented as total number of cells attached to the plate per view field (average of three fields). (**e**) Apoptosis of COS-7 cells with expression of α_v_β_3_ (COS7αVβ3) or β_3_ (COS7β3) treated by 5 μM ProAgio. Cell apoptosis is presented as % apoptosis by defining the apoptosis of untreated cells as reference 0%. Error bars in **a**,**d** and **e** are s.d. from measurement of five independent experiments. NS *P*>0.05, **P<0.005, ***P<0.001 calculated by unpaired two-tailed Student's *t*-test. NS, not significant.

Due to unique expression patterns and frequent disease association, several pairs of integrin are promising targets for the development of therapeutics for a number of pathological conditions^15^. Most approaches focus on the integrin ligand binding. However, significant drawbacks, such as the effect of activation of integrin signalling by ligand binding limit the clinical success of the integrin ligand antagonist. Most recently, Van Agthoven *et al*.^23^ reported a novel interaction of a variant of physiologic ligand fibronectin with integrin β_3_. The interaction did not lead to conformation changes resulting from ligand binding, thus consequentially activating integrin outside-in signalling, which provides a novel concept for developing integrin ligand-based antagonist. Our approach does not target ligand binding (Fig. 6b). In fact, ProAgio induced HUVEC apoptosis with or without ligand binding, and ProAgio binding did not interfere with RGD binding (Figs 6a and 2a), suggesting that the effects of ProAgio are independent of ligand binding. Effective induction of cell apoptosis through integrin α_v_β_3_ via a mechanism different from anoikis is a unique feature of ProAgio. This feature may bring a very important advantage in future potential clinical applications, as ProAgio does not need to compete with strong integrin–ligand interactions, and does not need to induce anoikis that may not occur in actual situations due to the fact that the cell-to-ECM adhesions always involve interactions between multiple pairs of integrin and multiple types of ECM. It is intriguing that ProAgio binding triggers recruiting and activating caspase 8 at cytoplasmic domain of β_3_, a mechanism of apoptosis induction similar to that was observed by Stupack *et al*.^25^ It is speculated that IMD would occur during tissue remodelling to remove excess unwanted cells. Our study further suggests that this apoptosis mechanism can actually be induced by a molecular interaction involved in the allosteric βA domain of β_3_ integrin. This is important as the notion provides a novel concept to develop integrin α_v_β_3_ target therapies that would be able to directly remove disease causative and integrin α_v_β_3_-expressing cells regardless of ligated or unligated integrin. Integrin β_3_ knock-out allow developing larger tumour with more vessels. Since the activity of ProAgio is dependent on the presence of high levels of integrin α_v_β_3_, therefore, it would be expected that ProAgio will not be effective in integrin β_3_ knock-out mouse.

The βA-groove is very close to the RGD-binding site in the βA domain. An open question is how the ProAgio–integrin interaction at the groove triggers effects that are so different from those of RGD binding. One plausible speculation is that ProAgio-binding to the βA-groove induces conformation changes that are different from that of RGD ligand binding, which consequently trigger different signalling that allows caspase 8 recruiting to the cytoplasmic domain of β_3_. Indeed, previous structural study of the integrin–RGD complex showed that RGD interacts with a site formed by ‘D3-A3' and ‘D4-A4' loops of β-propeller domain of α_v_ integrin. The Aspartate of RGD interacts with βA domain of β_3_ integrin, where the carboxylate side chain of the Asp protrudes into a cleft formed by βA ‘A-α2' and ‘C-α3' loops. These interactions are critical for an inward conformational change of the βA domain and the consequential integrin signalling^22^,^28^. However, our modelling, crosslinking and mutations demonstrate that ProAgio does not contact these RGD interacting elements. Instead, ProAgio contacts α2 helix, ‘B-C' loop and ‘α2-α3' loop (Figs 2e and 6b), which are not involved in RGD binding. An ultimate answer to the question may require a comprehensive structural study to understand the differences in structure/conformation changes on ProAgio binding and RGD ligand binding.

In our modelling, ProAgio only contacts β_3_. It is intriguing why ProAgio has a specificity for α_v_β_3_. Careful examination of X-ray crystal structures of α_v_β_3_ and α_IIb_β_3_, the other authentic α subunit that paired with β_3_, reveals that the ProAgio-binding site, the β-groove, is different in both cases (Supplementary Fig. 8). The key contact residues K, K and Q locate in a region of α1 α-helix in α_v_β_3_, while the same region forms a loop in α_IIb_β_3_. The relative orientation of α1 and α2 helixes are important for the ProAgio binding (from our docking and modelling), while these two helixes are pushed far apart in α_IIb_β_3_ (Supplementary Fig. 8). Thus, ProAgio can only binds to α_v_β_3_ but not α_IIb_β_3_ as shown in our binding and cell attachment studies. Apparently, the fact that ProAgio interacts with α_v_β_3_ but not α_IIb_β_3_ provides another support for the ProAgio and α_v_β_3_ interaction site. Interestingly, COS-7 cells expressing β_3_ (without α_v_) were able to attach to ProAgio. ProAgio was also able to less effectively induce apoptosis of the β_3_-expressing COS-7 cells (Fig. 6c–e). These results provide an additional support for our design that ProAgio mainly contacts with β_3_.

## Methods

### Reagents, antibodies, cell lines and protein expression/purifications

Cell lines PC-3, 4T-1, CHO, HEK299 and COS-7 were purchased from ATCC, and HUVEC cells were purchased from Invitrogen. The cells were cultured by following the vendor's instructions. Caspase 8 Inhibitor II, Caspase 9 Inhibitor III, Cilengitide, Integrin pairs, α_v_β_3_, α_1_β_1_ and α_IIb_β_3_, Rhodamine Phalloidin, BS2G and glutraldehyde were purchased from EMD Millipore, Billerica, Roche R&D, Invitrogen and Sigma-Aldrich, respectively. Avastin was a prescription drug purchased from Walgreens. Endostar was purchased from SIMCERE China. PEGylation agent Maleimide-20K for protein Cys pegylation was purchased from JenKem Technology. Antibodies against β3-integrin (AB2984), α_v_-integrin (#4711), α_IIb_-integrin (D8V7H), FAK (#3285), Phospho-FAK (#3283), Caspase 8 (1C12), Cleaved Caspase 8 (18C8), Cleaved Caspase 9 (#9505), Cleaved Caspase 3 (#9661), Cleaved Caspase 7 (#9491), mouse CD31 (561813), vinculin (V9131) and GAPDH (365062) were purchased from Millipore, Cell Signaling, Danvers, Abcam and Santa Cruz Biotechnology, respectively. Antibody against ProAgio was raised using recombinant ProAgio expressed/purified from *E. coli* as an antigen. The complementary DNAs (cDNAs) that encode D1-CD2 was adapted from our previous studies^20^. The cDNAs for D1-CD2 and variants were subcloned into bacterial expression vector pGEX-2T. The recombinant proteins were purified from bacterial lysates by a two column procedure established in our laboratories. The cDNAs encode for integrins α_v_, α_IIb_ and β_3_ were purchased from Addgene. The cDNAs were subcloned into a mammalian expression vector pCDNA3.1 for exogenous expression of different integrins in cultured cells.

### Computation modelling/docking

Integrin coordinates were taken from the RCSB Protein Data Bank (ID: 1L5G, 4MMZ)^29^. The domain 1 of cell adhesion protein D1–CD2 (Protein Data Bank ID: 1HNF)^30^ was used as starting point for docking.

The docking was performed with HADDOCK 1.2, supporting docking to make use of experimental information to drive the docking while allowing for various degrees of flexibility^31^. D1-CD2 was first randomly docked into βA domain of β_3_. For comparison, three different variants were also randomly docked into βA domain of β3. To further refine the binding of integrin and ProAgio, ProAgio (variant 3) was docked into integrins α_v_β_3_ together with our crosslinking-drive refinements. The active residues used to define the ambiguous interaction restraints (AIRs) were those showing the direct crosslinking contacts and were solvent accessible. The surface accessibility data were calculated with the program NACCESS. Only the residues having either backbone or side-chain relative solvent accessibility above 40% were retained for the definition of active and passive residues. The passive residues correspond to the residues that are surface neighbours of the active residues as identified by visual inspection and that have a high level of solvent accessibility (40%). A total of 1,000 conformers of the integrin with ProAgio or D1–CD2 complex were generated using only AIRs, van der Waals energy and electrostatic terms in crystallography and NMR System (CNS). The 200 conformers with lowest molecular energies were subsequently subjected to semi-flexible simulated annealing and refinement with explicit water and only backbone restraints for residues outside the interface. The solutions were clustered using an arbitrary root-mean-square deviation (RMSD) and ranked according to their intermolecular interaction energy (sum of Van der Waals, *E*_elec_; electrostatic, *E*_vdw_ and AIRs energy terms, *E*_AIR_). The lowest energy structures of each cluster were selected for the analysis.

*ELISA binding*. The commercial recombinant integrins α_1_β_1_, α_v_β_3_ and α_IIb_β_3_ were immobilized on ELISA plates. Various concentration of ProAgio were added into the plates. In some cases, polyLys was co-added into the plates at a final concentration of 3 mM. After incubation and extensive washing, antibody that recognizes ProAgio was added into mixture. The plates were incubated at room temperature for 4 h. Excessive antibody was removed by washing. The bound ProAgio was quantified by secondary antibody that recognizes anti-ProAgio antibody. The binding data were plotted and analysed via Scotchard analyses to deduce the Kd and binding stoichiometry.

*SPR*. The commercial recombinant integrins α_1_β_1_, α_v_β_3_ and α_IIb_β_3_ were diluted to a concentration of 100 μg ml^−1^ and immobilized on to the CM5 chip with response units around 500. Then PEGylated ProAgio bindings were carried out at various concentrations with the running buffer of 1 × HBSS 10 mM HEPES, 150 mM NaCl, 5 mM MgCl_2_, pH7.4. The flow rate of 50 μl min^−1^, and the binding time was 12 min. Kds were deduced by calculation via binding curves of various concentrations.

### Nude mice xenograft and treatments, and toxicity

All animal experiments were carried out in accordance with the guidelines of Institutional Animal Care and Use Committee (IACUC) of Georgia State University. Six-week-old male/female nude mice or Balb/c mice were subcutaneously injected with 5 × 10^6^ of PC-3 or 4 T-1 cells. Tumour formation and volumes were assessed every 2–4 days. Tumour volumes were measured by two perpendicular diameters of the tumours with the formula ½ (length) × (width)^2^. The tumour-bearing mice were subjected to intraperitoneal injections of appropriate agents at indicated frequencies. The treatments started 5 days post tumour inoculations or till tumour reached the average size of 230 mm^3^. The tumours were collected and weighed at the end of the experiments. Tissue sections were prepared from collected tumours or other organs. For toxicity studies, 6-week-old healthy male healthy CD-1 mice were intravenously injected with appropriate agents at indicated frequencies and doses. The animals were returned to their cages after injection. The behaviours of animals were closely monitored. Blood samples were collected at appropriate time points. For analyses of urine albumin, animals were placed in metabolic cages. Urine samples were collected in 24 h. At the end of experiments, animals were killed. Tissue samples from various organs were collected for tissue sections. The tissue sections were analysed by immunofluorescence or hematoxylin and eosin stains using commercially available antibodies as indicated. Statistical analyses were done in comparison with the control group with unpaired two-tailed Student's *t*-test.

### Determination the integrin binding site by chemical crosslinking followed by MS analyses

Crosslinking by BS2G (Supplementary Fig. 3a). Recombinant integrin α_v_β_3_ were mixed with his-tagged ProAgio in 1:0.8 ratio. After 40 min incubation, the crosslinking agents BS2G were added to the mixture in 40-folds molar excess. After crosslinking reaction for 150 min, the crosslink mixture was denatured and separated by 5% SDS–PAGE. Crosslinking of ProAgio to the integrin was verified by immunoblot using antibodies against ProAgio and integrin β_3_ (Supplementary Fig. 3b). The identified crosslinking bands were sliced out and subjected to trypsin digestion followed by LC-MS analyses. The crosslinked peptides were identified by the procedure similar to that described by Bing Yang *et al*.^32^ using pLink analyses. The identified crosslinking peptides were then analysed by MS/MS to determine the aa sequence of the peptides. Several crosslinked peaks were identified (Fig. 2c and Supplementary Fig. 3e, sequence derived from MS/MS is shown on top). Many were crosslinked between integrins (Supplementary Fig. 3e, as examples). The ProAgio–integrin crosslink was observed in three independent repeating crosslinking experiments. Supplementary Table 2 list all relevant peptide crosslinks by BS2G.

Crosslinking by glutaraldhyde (Supplementary Fig. 3A). His-ProAgio was mixed with recombinant integrin α_v_β_3_ in 0.7:1 ratio in HEPES buffer. The freshly prepared glutaraldhyde was added into the mixture in three folds of molar excess. After 1 h incubation on ice, the crosslinking was stopped by adding 1 M Tris-HCl buffer, pH=7.4. The crosslinking mixture were separated by electrophoresis and Gel-code staining (Supplementary Fig. 3c, right panel). The crosslinking mixture was then denatured by 8 M urea. The crosslinked His-ProAgio and the integrin were pulled down by Ni-NTA beads. The precipitations were analysed by SDS–PAGE followed by immunoblot using antibody against integrin β_3_ (Supplementary Fig. 3c, left panel). Clearly, His-ProAgio crosslinked to integrin β_3_ (Supplementary Fig. 3c). After elution from the Ni-NTA beads, precipitates were dialysed against HEPES buffer pH=6.9. The recovered ProAgio–integrin crosslinks were digested by trypsin and subsequently analysed by matrix-assisted laser desorption/ionization (MALDI)-MS. An identical procedure was performed without the addition of crosslinking agent glutaraldehyde and without Ni-NTA beads pull-down. Peptide crosslinks were identified manually. Crosslinking experiments were performed more than three times. The resultant mass spectrum with/without the addition of glutaraldehyde and pull-down were closely compared. Control peaks were verified and all the new crosslinked peaks were analysed for the peptides. Several peptide MS peaks were detected in the glutaraldehyde crosslinked mixture while they were absent in non-crosslinked mixture. An identical MS peak at 2907.9 Da was detected in three independent repeating crosslink experiments. This peak was further subjected to MALDI-MS/MS analyses to identify the crosslinked peptide (Fig. 2c, sequence derived from ms/ms and manual analysis is shown on top).

All reversed-phase high-performance liquid chromatography (RP-HPLC)−MS/MS experiments were performed on a LTQ-Orbitrap Elite mass spectrometer equipped with EASY-spray source and nano-LC UltiMate 3000 high-performance liquid chromatography system.

### Endothelial tube formation assays

Endothelial tube formations were carried out with the endothelial tube kit (Invitrogen). Briefly, HUVEC cells were seeded in culture plate coated with Geltrex. After 30 min incubation, agents, for example, FBS, proteins and other agents, were added to the HUVECs. The cells were further cultured for additional 16 h. The formed endothelial tubes were analysed under light microscope and quantified.

### Cell attachment assays

The cells were cultured overnight under standard conditions. Next day, different cells (with appropriate cell numbers) were transferred to a new plate with wells that were coated with different proteins (indicated in the figures) with fresh medium with the addition of appropriate agents (indicated in the figures). The cells were further cultured for 35 min and washed three times gently. The attached cells were either directly counted. In all attachment assays, the attachment is presented as total number of cells attached to the plate per microscopic view field (calculated from average of three view fields).

### Apoptosis assays

For determination of EC_50_ of ProAgio and the effects of caspase 8/9 inhibitors, HUVEC cells were cultured overnight. The cells were shifted to fresh medium next morning. Various concentrations of ProAgio or D1-CD2, or caspase 8/9 inhibitors with 5 μM ProAgio were added into culture medium. After 10 h incubation, the apoptosis was determined using an apoptosis kit.

For all other apoptosis measurements, appropriate cells were cultured overnight. The cells were shifted to fresh medium next morning. Appropriate agents were added into the medium to appropriate concentrations. After 24 h incubation, the cell apoptosis was determined by cell counting using untreated cells as a reference. The cell counting was repeated five times in each experiment.

### Immunofluorescence staining

HUVEC cells were grown on glass coverslips coated with fibronectin for immunofluorescence microscopy. After treatment with proteins, cells were fixed with cold (+4 °C) 4% PFA in PBS (UBS) for 15 min and blocked by incubating with 3% BSA/PBS at 37 °C for 1 h. Vinculin antibody (1:1,000 dilution) was incubated with the coverslips for 45 min at 37 °C. The cells were washed with PBS for 10 min at room temperature before incubating with a 1:500 dilution of Alexa-488 conjugated secondary antibody. The cells were then incubated with Phalloidin conjugated rhodamine for 30 min at 37 °C Cells were mounted with Prolong-Gold antifade reagent that contained DAPI (Invitrogen, Carlsbad). For immunofluorescence staining of tissue sections, snap frozen tissue blocks were sliced into 10 μM thick sections using a cryostat. The procedure similar to that used in immunostaining of fixed cells was used for tissue section immunofluorescence staining.

*Vessel quantitation*. Sections from tumours or various organs of mice that were treated by appropriate agents were stained using an anti-mouse CD31 antibody and a green fluorescence-conjugated secondary antibody. After the highest vessel density within a section was determined under lower objective lens ( × 5), an area of 319.8 × 319.8 μM with the highest amount of vessels was scanned under a × 20 objective lens and × 10 ocular lens. The CD31 immunofluorescence staining was quantified by manual counting using Imaging-J. Vessel Length is the total number of vessels length within each of the image was measured using a Zeiss LSM image browser. Mean Vessel Density was determined using methods described by Weidner *et al*.^33^ Briefly, any clear pattern of fluorescent stained or a cluster within the tumour tissue that separated from connective tissues was counted as one vessel. Microvessels in sclerotic areas or adjacent to connective tissues were not considered. Branch points was determined by manually counting the number of branch points under the microscopic view. Any junction of two lines of CD31 stains counts as one branch point. All data were shown in 319.8 × 319.8 μm^2^ area of view field and are the average of repeating three view fields per section. Four sections from each tumour/organ were counted.

### Statistical calculations

Data were statistically analysed by comparing two appropriate groups. The *P* values were calculated using unpaired two-tailed Student's *t*-test. In all figures and tables, NS means *P*>0.05 and statistically insignificant, *P<0.05, **P<0.01, and ***P<0.001.

## Additional information

**How to cite this article**: Turaga, R. C. *et al*. Rational design of a protein that binds integrin avß3 outside the ligand binding site. *Nat. Commun.* 7:11675 doi: 10.1038/ncomms11675 (2016).

## Supplementary Material

Supplementary Information Supplementary Figures 1-11 and Supplementary Tables 1-2
