# Supplementary material for: Rational design of a protein that binds integrin αvβ3 outside the ligand binding site
Source: Nat Commun. 2016 May 31;7:11675. doi: 10.1038/ncomms11675 (PMC4895024; doi:10.1038/ncomms11675)
Supplement: Supplementary Information — Supplementary Figures 1-11 and Supplementary Tables 1-2 [file ncomms11675-s1.pdf]

Turaga et al., Supplemetary Figure 1

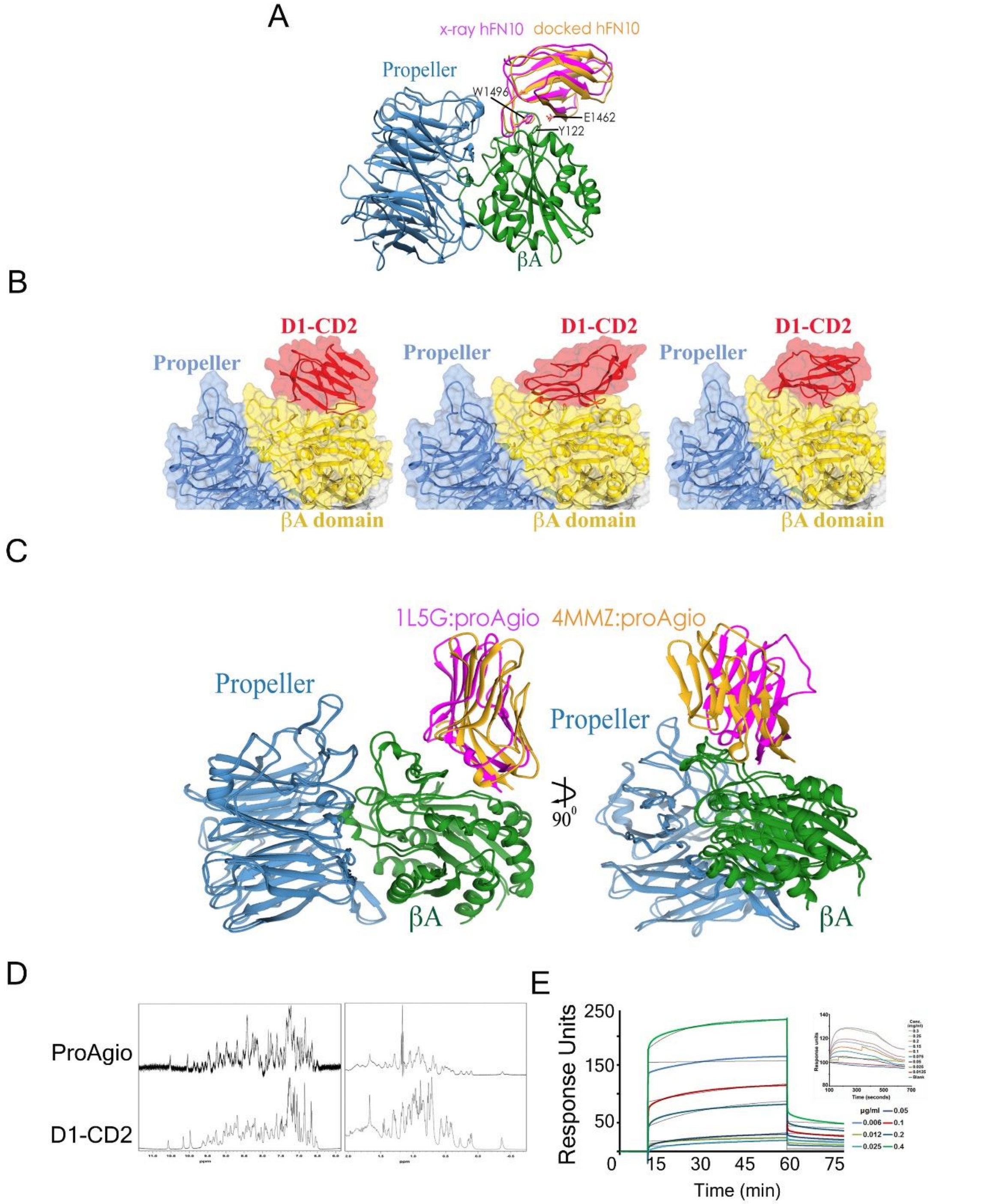

Supplementary Figure 1 (A), (B), and **(C)** Docking of a physiologic ligand of integrin $\alpha_v\beta_3$, the tenth type III RGD domain of wild-type fibronectin **(A)**, D1-CD2 **(B)**, and the D1-CD2 variant 3 **(C)** to βA-domain of integrin $\alpha_v\beta_3$ using software HADDOCK 1.2. In **(A)**, the yellow colored RGD domain is the docking model, while the purple colored RGD domain is crystal structure by Van Agthoven, J. F. and co-workers. In **(B)**, different models in various different orientations of D1-CD2 docking at βA groove are shown. In **(C)**, purple color shows ProAgio docking to the crystal structure 1L5G, while yellow color show D1-CD2 variant 3 docking to the crystal structure 4MMZ. Left and right images are same docking model with 90° twist to show the difference in orientation of 1L5G and 4MMZ dockings (yellow and purple). **(D)** ^{1}H-NMR spectra of ProAgio and D1-CD2 were recorded using Varian Inova 600 MHz spectrometer. Protein samples were prepared by dilution with 10 mM Tris and 10% D_2O pH = 7.4 to final protein concentration of 200 μM. **(E)** The representative binding curves of binding of PEGylated ProAgio **(Left)** or D1-CD2 **(Right, and smaller panel)** to integrin $\alpha_v\beta_3$ were monitored by SPR with integrin that were immobilized on the Biacore chip. Thin black lines in left panel are the fitting curves based on one-to-one binding mode.

Turaga et al., Supplementary Figure 2

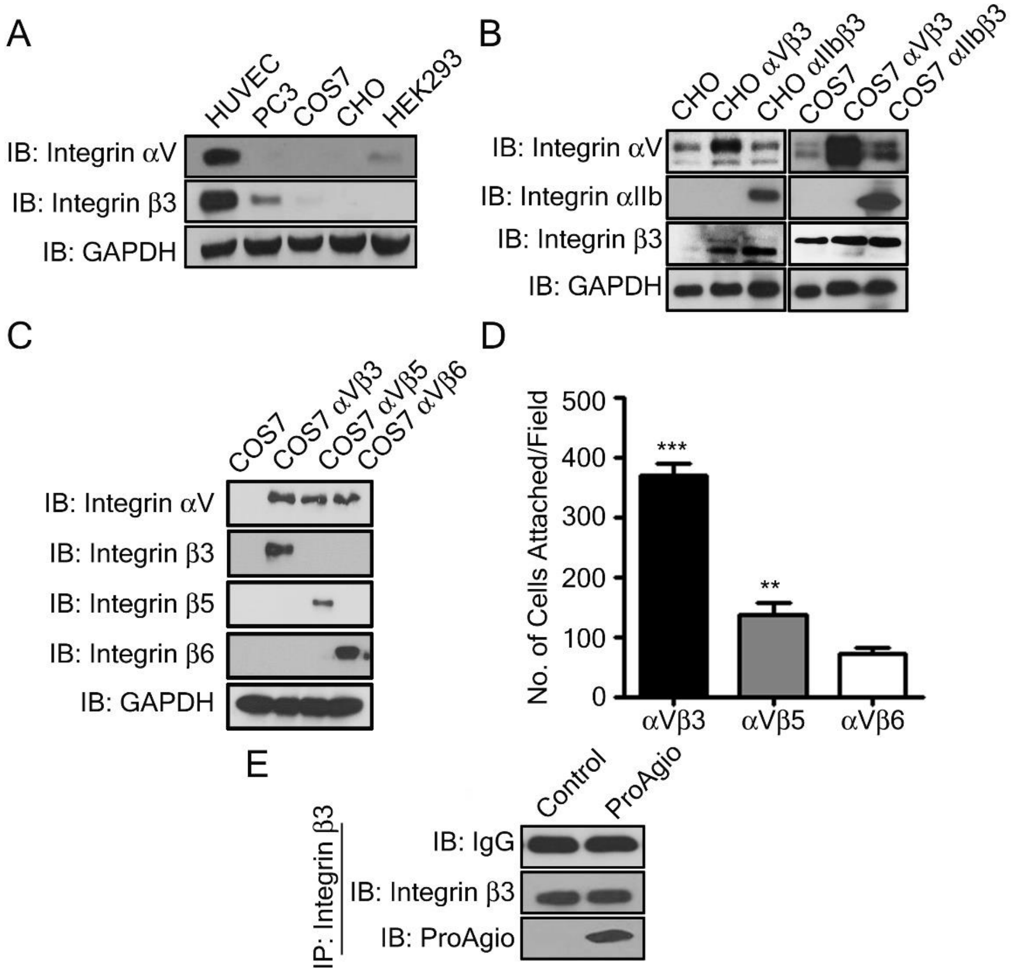

Supplementary Figure 2 (A) & **(B)** The levels of endogenous and exogenous integrin αV(IB: Integrin αV), β_3 (IB: Integrin β3), and αIIb (IB: Integrin αIIb) of indicated cells were analyzed by immunoblot of cell lysates. CHOαVβ3, CHOαIIbβ3, COS7αVβ3, and COS7αIIbβ3 are derived from CHO and COS7 cells with exogenous expression of $\alpha_v\beta_3$ and $\alpha_{IIb}\beta_3$ respectively. **(C)** The levels of exogenous expressed integrin αV (Integrin αV), β3 (Integrin β3), β3 (Integrin β5), and β_6 (Integrin β6) in COS-7 cells were analyzed by immunoblot of cell lysates from COS7αVβ3, COS7αVβ5, and COS7αVβ6 respectively. **(D)** Attachment of COS7 cells with exogenous expression of integrin αvβ3, αvβ5, and αvβ6 to plate coated with ProAgio. The attachment presented as number of cells attached per microscopic view field. Error bars are standard deviations from measurement of five independent experiments. **(E)** Co-immunoprecipitation of integrin β_3 with ProAgio (IP: Integrin β3) from the HUVEC cell lysate was examined by immunoblot of ProAgio (IB: ProAgio). The cells were treated with 5 μM ProAgio for 6 hours prior to the extracts preparation. IgG indicate the amounts antibody used in IP. Immunoblot of integrin β_3 (IB:Integrin β3) indicates amount of β_3 was precipitated down in the co-IPs. Immunoblot of GAPDH in **(A)**, **(B)**, and **(C)** are loading controls.

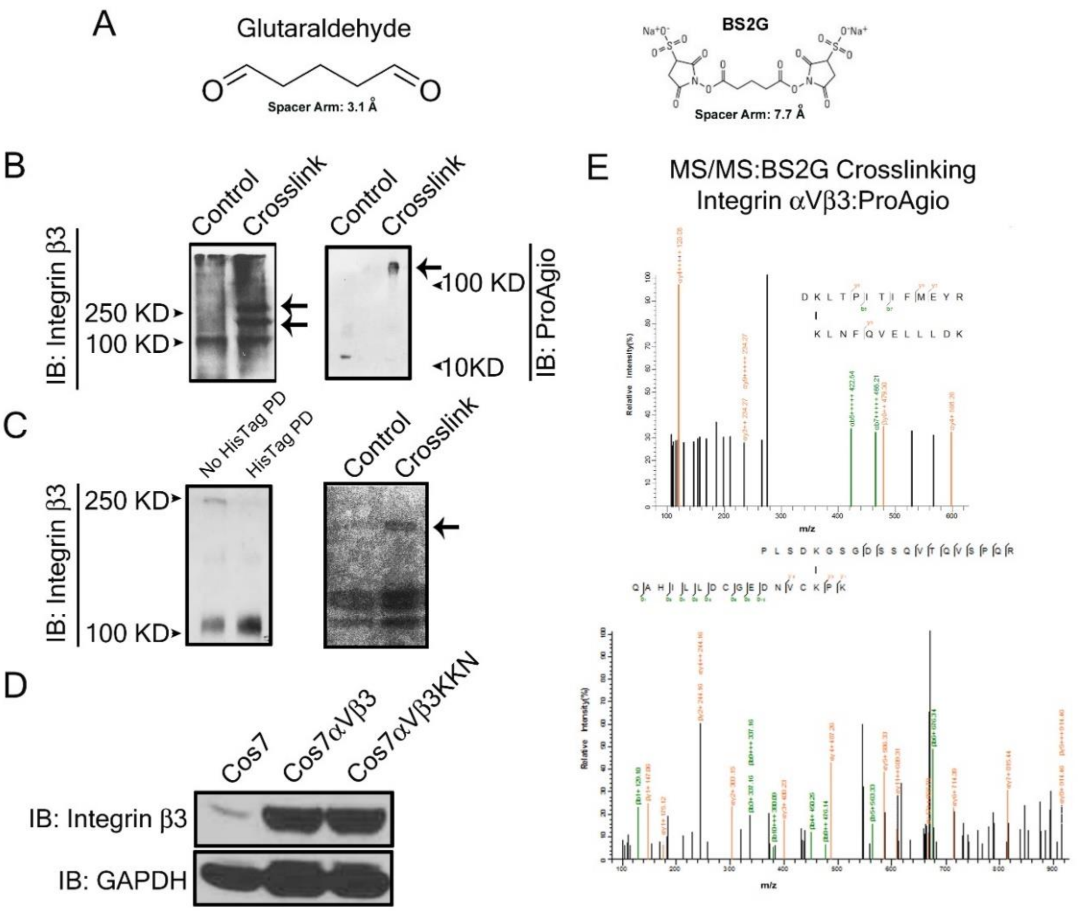

Supplementary Figure 3 (A) Structure drawing of crosslinking agents BS2G and glutaraldhyde. **(B)** Crosslink of His-ProAgio with recombinant integrin $\alpha_v\beta_3$ using BS2G were examined by immunoblots of integrin β_3 (IB: β3 Integrin) **(Left)** and ProAgio (IB:ProAgio) **(Right)**. The controls in both left and right panels are the ProAgio-integrin complex without crosslinking by BS2G. **(C)** Crosslinks of His-ProAgio with recombinant integrin $\alpha_v\beta_3$ using glutaraldhyde were examined by Gel-code staining **(Right)** and immunoblots of integrin β_3 (IB: β3 Integrin) **(Left)**. Control in right panel is the ProAgio-integrin complex without crosslinking by glutaraldhyde. Crosslinking mixture were separated by denaturing and His-pull down (His tag PD) or without pull-down (No HisTag PD) prior to electrophoresis and immunoblot. In **(B)** and **(C)**, arrows indicate the ProAgio-integrin crosslinks. The crosslinked bands were sliced out and subjected to trypsin digestion and followed M/S analyses. **(D)** Expression of integrin $\alpha_v\beta_3$ wild type (COS7$\alpha_v\beta_3$) and β3-KKN mutant (COS7$\alpha_v\beta_3$ KKN) in COS7 cells were examined by immunoblots of integrin β_3 (IB:Integrin β3). Immunoblot of GAPDH (IB:GAPDH) is a loading control. **(E)** Call-out regions ms/ms (top, ProAgio-integrin crosslinking, middle and bottom, integrin-integrin crosslinking) of the MS spectrum (*MS2*) of peptide fragments resultant from trypsin digestion of crosslinks of His-ProAgio with integrin $\alpha_v\beta_3$ using BS2G. The sequence on top is derived sequences using pLink based on ms/ms of the crosslinked peaks.

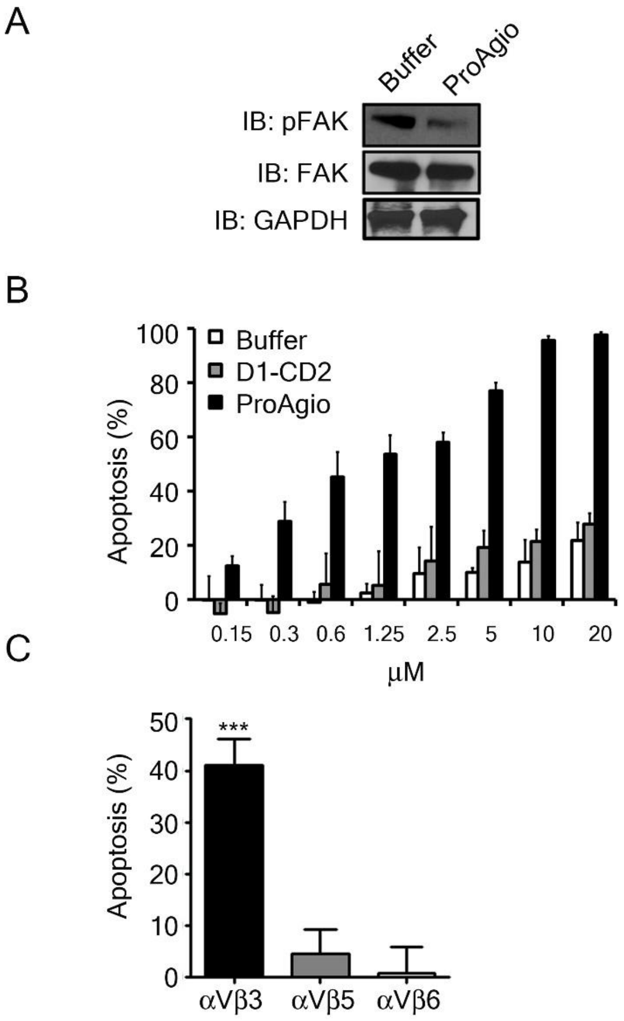

Supplementary Figure 4 (A) Cellular levels of phosphorylated FAK in HUVEC cells were examined by immunoblot (IB: pFAK). The cells were treated by indicated agents. Immunoblot of FAK (IB: FAK) indicates the levels of total cellular FAK. Immunoblot of GAPDH is a loading control. **(B)** Apoptosis of HUVEC cells was measured by apoptosis kit 10 hours after treatment with different concentrations of ProAgio or D1-CD2. Cell apoptosis is presented as Apoptosis (%) by defining the apoptosis of untreated cells as reference 0% apoptosis. **(C)** Apoptosis of COS7 cells with exogenous expression of integrin αvβ3, αvβ5, and αvβ6 in the presence of 3 μM ProAgio was measured by cell apoptosis kit. Cell apoptosis is presented as Apoptosis (%) by defining the apoptosis of untreated cells as reference 0% apoptosis. Error bars in **(B)** and **(C)** are standard deviations from measurement of five independent experiments.

Turaga et al., Supplementary Figure 5

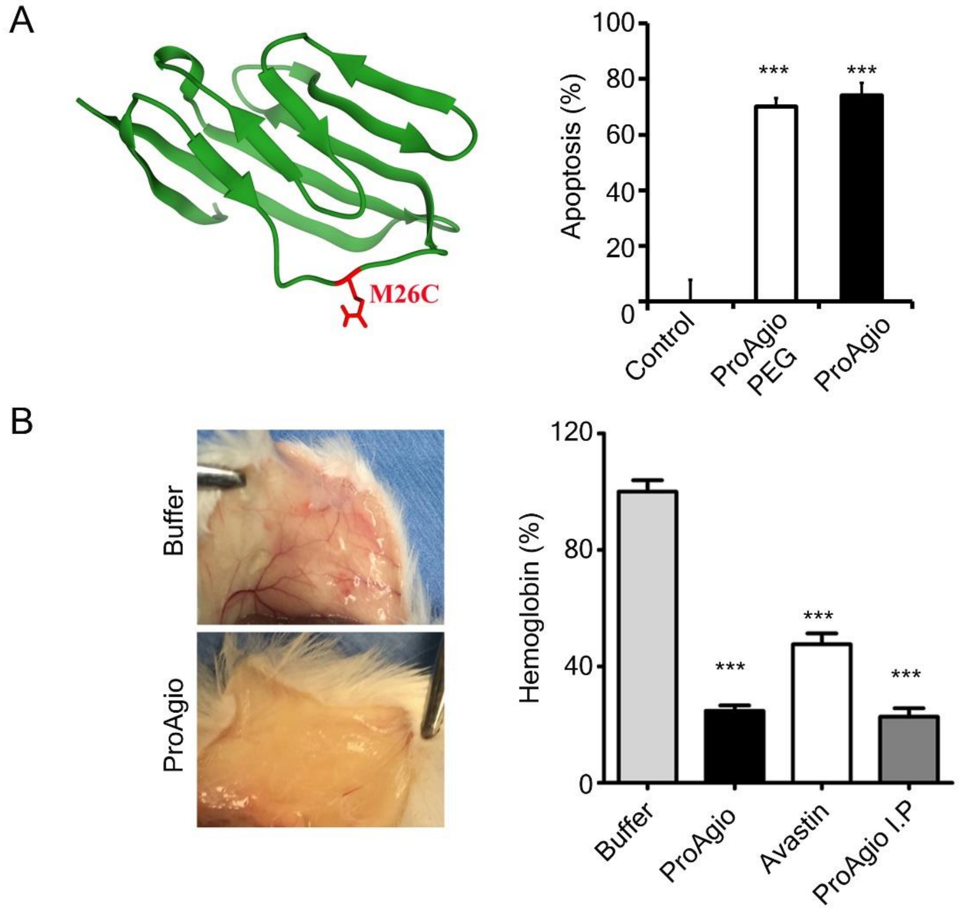

Supplementary Figure 5 (A) (Left) Model structure of ProAgio. Red color indicates the position of introduced Cys residue for PEGylation. **(Right)** Apoptosis of HUVEC cells was measured by cell counting 24 hours after treatment with buffer, ProAgio, or ProAgio-PEG. Cell apoptosis is presented as % apoptosis by defining the apoptosis of untreated cells as 0%. (B) Matrix-gel plug assay, 400 μl of matrix-gel in mixing with indicated agents was s.c. implanted on the right flank of Balb/c mice. The matrix-gel Geltrex^{TM} was pre-mixed with other components suggested by vendor before addition of the indicated agents. After two weeks, pictures of the matrix-gel insertion site were taken. (**Left)** are representative images of the matrix-gel plugs in mix with either ProAgio or buffer saline. (**Right)**, quantitation of angiogenesis in implanted matrix-gels, the matrix-gels were retrieved from the mice. The hemoglobin contents in the matrix-gel plagues were analyzed by standard method, and presented as relative hemoglobin content by defining the hemoglobin levels of buffer gel plague as 100%. ProAgio IP means the ProAgio was administered by i.p. injection (5 mg/kg one dose every two days) instead of pre-mix with matrigel. Error bars are standard deviations from measurement of five independent experiments.

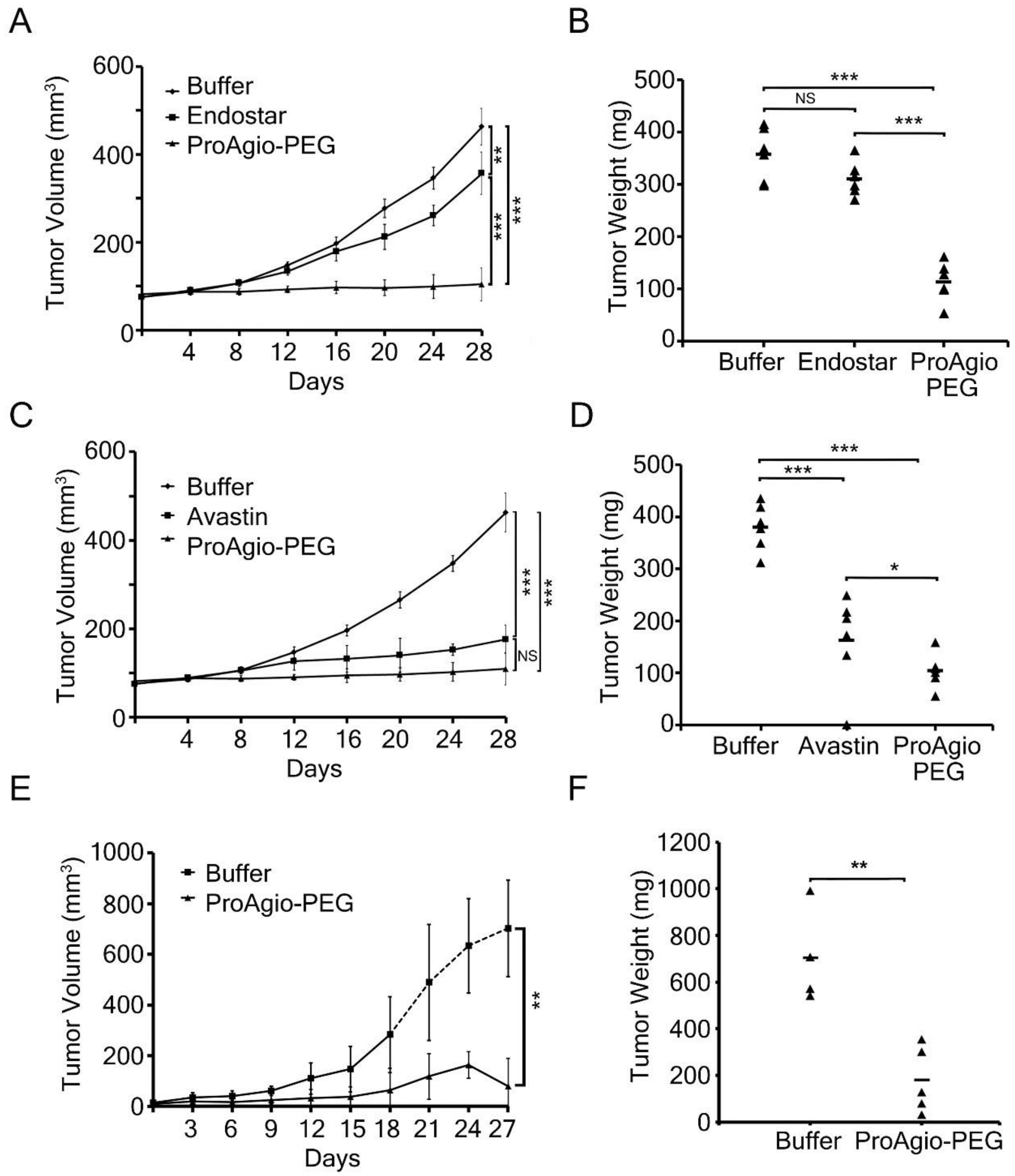

Supplementary Figure 6 Growth of xenograft PC-3 tumors **(A)**, **(B)**, **(C)**, and **(D)** or orthotopic 4T-1 tumors **(E)** and **(F)** under the treatment of indicated agents was monitored by; **(A), (C),** and **(E)** growth curve by measuring tumor volumes every four days, or **(B), (D),** and **(F)** endpoint weights of the harvested tumors. Treatments started 5 days post tumor inoculations. In **(F)**, one tumor completely disappeared in ProAgio treatment group. The dot line in **(E)** in the growth curve is due to extra-large size of tumors that led to pre-termination of experiment (per IACUC regulation). The pre-terminated tumors were not included in both growth curve and final tumor weights. Error bars are standard deviations from measurement of six mice.

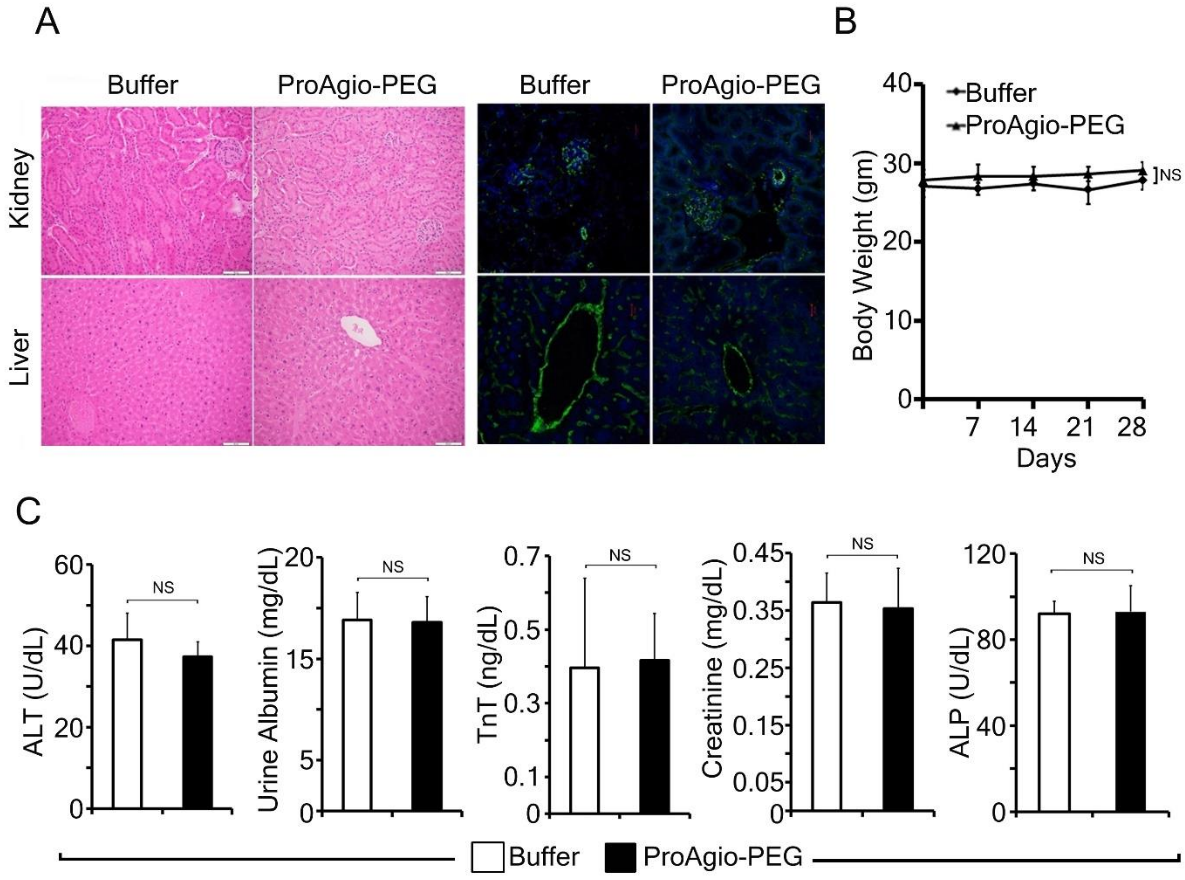

Supplementary Figure 7 (A) Represent images of Hematoxylin staining **(Left)** and IF staining using anti- mouse/CD31 antibody **(Right)** of tissue sections of kidney and liver collected from the mice that were treated with ProAgio-PEG 20 mg/kg (10 doses for 20 days) or buffer. The images are typical of stains of at least two view fields of four sections from each organs. **(B)** Body weights of tumor-bearing mice were measured every four days during the treatments with ProAgio-PEG 20 mg/kg (10 doses for 20 days) or buffer. **(C)** Plasma levels of ALT, ALP, Creatine, and TnT, and urine levels of albumin were measured using corresponding commercially available kits. Mouse blood and urine samples were collected from CD-1 mice 48 hours after treatment with three doses of 60 mg/kg ProAgio-PEG or buffer. Error bars in **(B)** and **(C)** standard deviations from measurement of six mice.

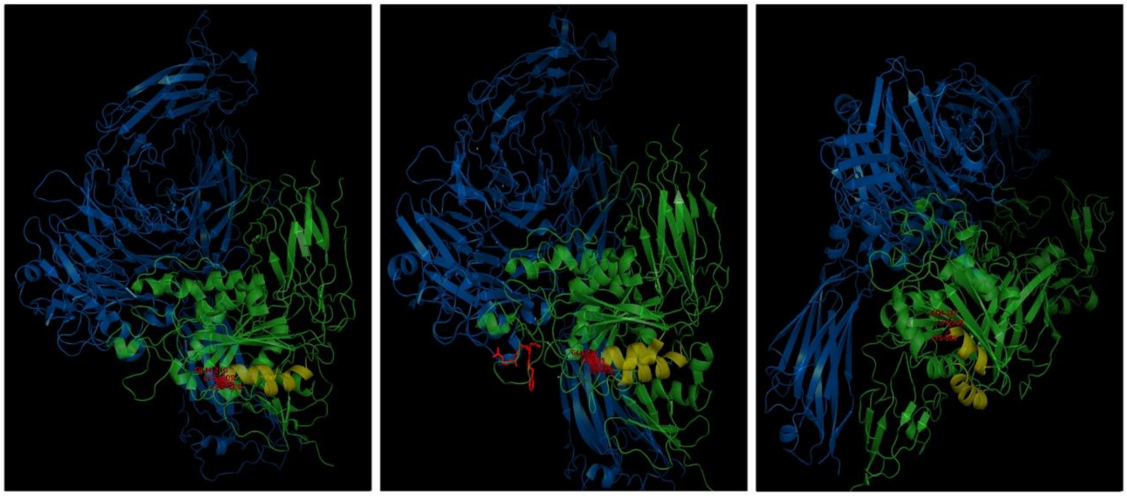

PDB ID: 1JV2 $\alpha V\beta 3$ Integrin | PDB ID: 1L5G $\alpha V\beta 3$ Integrin with RGD | PDB ID: 3FCS $\alpha IIb\beta 3$ Integrin

Supplementary Figure 8 Structure features of the β-groove and nearby regions of integrin $\alpha_V\beta_3$ with/without RGD binding **(Left and middle)** and $\alpha_{IIb}\beta_3$ **(Right)**. The structures in figures are adopted from the indicated PDB files in protein structure data bank.

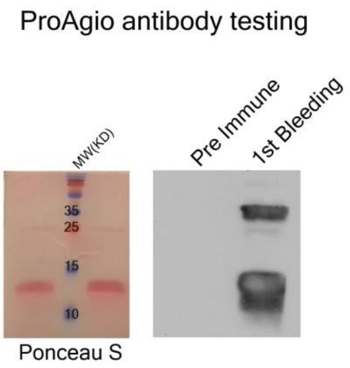

Supplementary Figure 9 Screening of the antibody generated against ProAgio in rabbit. Left panel indicate the ponceau staining, right panel indicate the corresponding western blot for pre immune and 1st bleed. 1:2000 dilution of generated ProAgio antibody was used to perform the western blot.

Turaga et al., Supplementary Figure 10

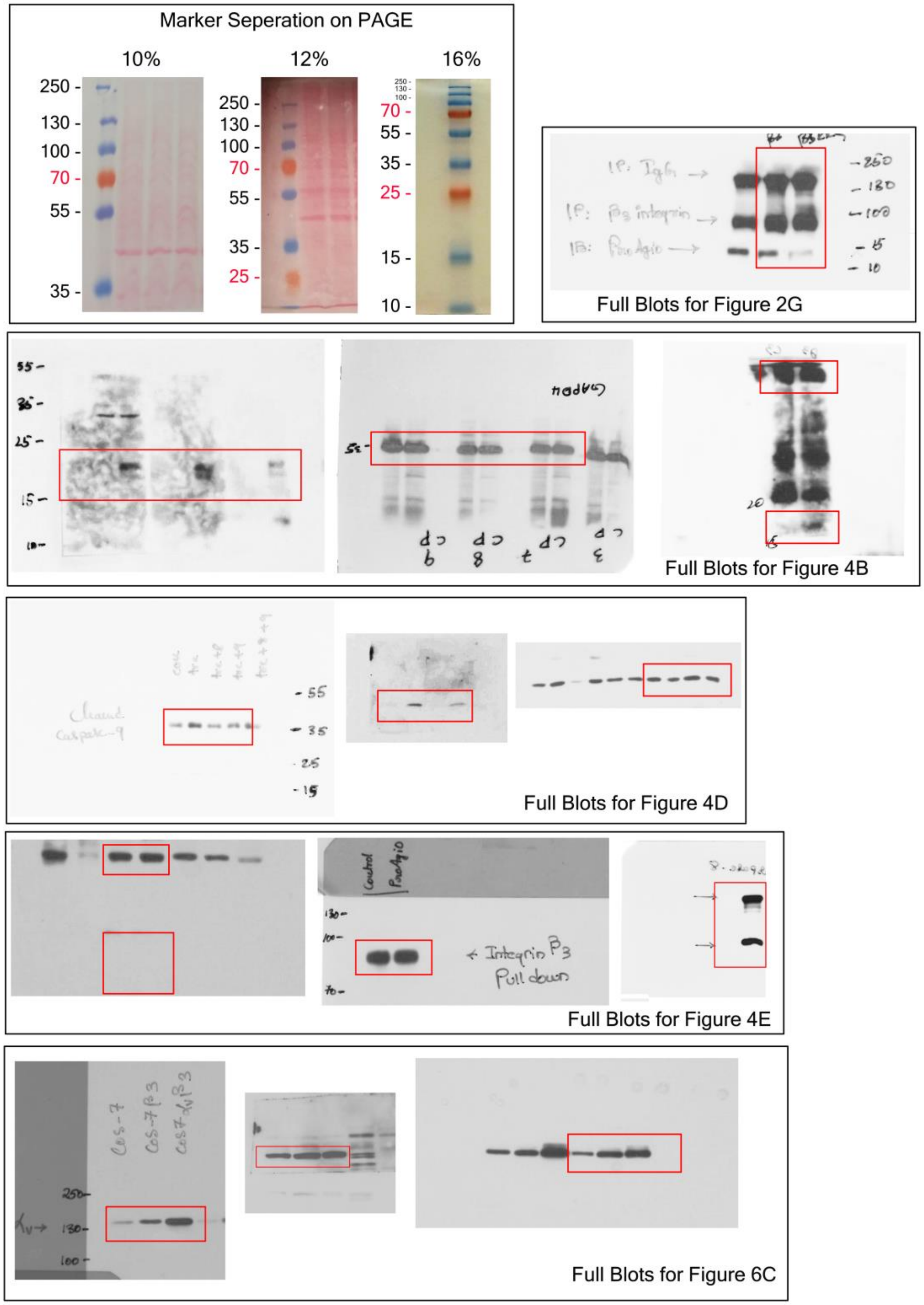

Supplementary Figure 10 Complete western blots of the main figure 2, figure 4 and figure 6

Turaga et al., Supplementary Figure 11

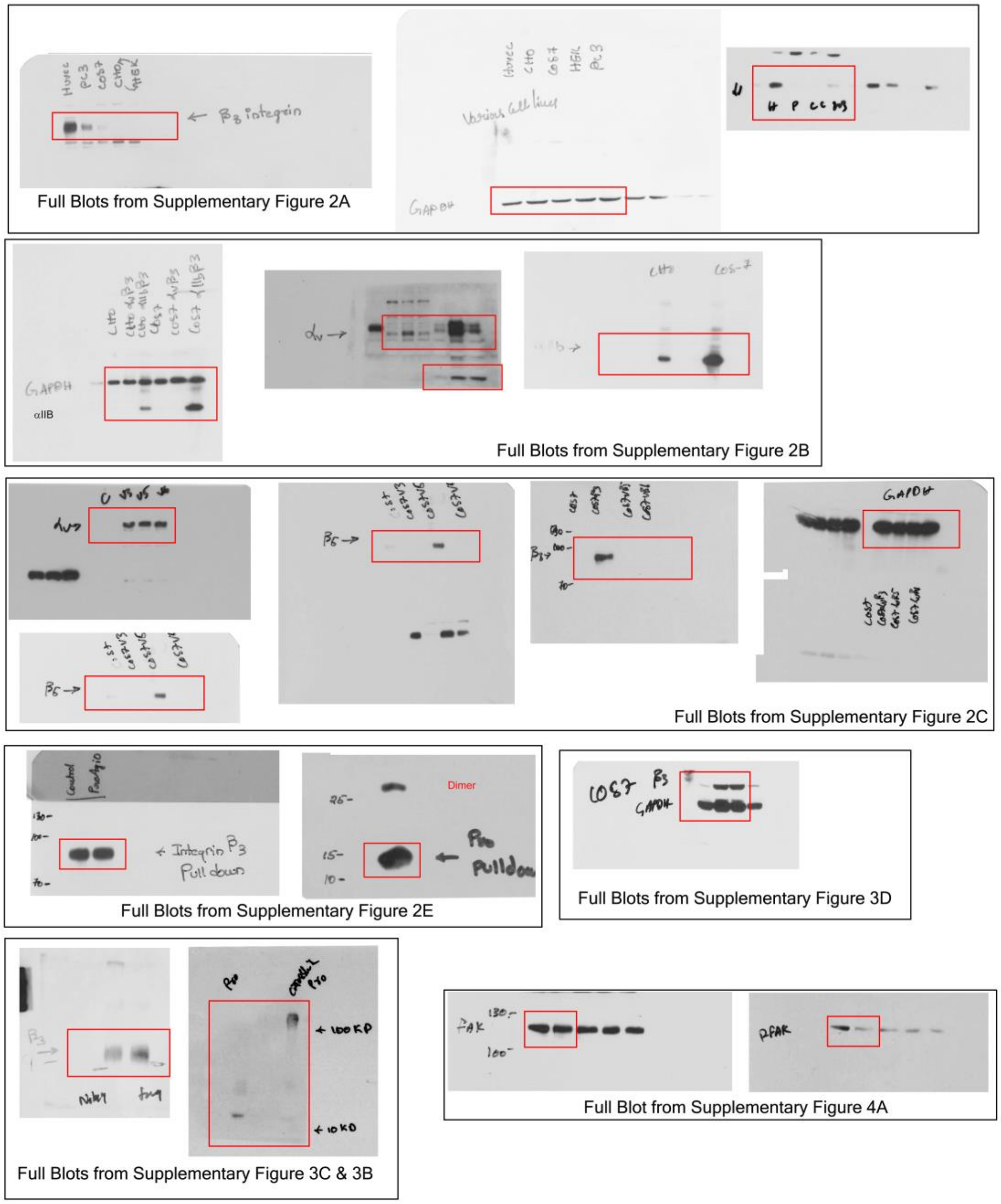

Supplementary Figure 11 Complete western blots of the supplementary figures 2, 3 and 4.

	Buffer	Endostar	Avastin	ProAgio
V-Length (μM)[#]	1328.6 +/- 157	1039 +/- 170*	564.6 +/- 145**	260 +/- 79***
MVD[##]	16.8 +/- 1.8	14.3 +/- 1.3*	8.5 +/- 2.1**	5.5 +/- 1.3**
BP	8.4 +/- 2.0	8.0 +/- 1.7*	2.3 +/- 1***	0.5 +/- 0.2***

Supplementary Table 1: CD31 Quantification of PC3 xenograft tumors treated with Buffer (PBS), Endostar, Avastin and ProAgio (n = 6). 5 sections from each tumor was stained and quantified. Quantification was performed for vessel length, mean vessel density (MVD) and branch points (BP) using ImageJ software.

	Mass (Da)	Peptides	Regions	δ ppm
1	1225.6321	KFDR-KFDR	αVβ3(619) - αVβ3(619)	1.63
2	2233.2514	IRSKVELEVR-KEKETFK	αVβ3 (354) - ProAgio(50)	1.66
3	1283.7811	QKGAIR - LKQK	αVβ3 (505) - αVβ3(503)	1.41
4	4663.2408	VLEDRPLSDKGSGDSSQVTQVSPQR-QAHILLDCGEDNVCKPK	αVβ3(72) - αVβ3(603)	6.82
5	2037.9879	WEKTSDKK-TEMKQER	ProAgio(42) - αVβ3(119)	1.47
6	3181.7315	DKLTPITIFMEYR-KLNFQVELLLDK	αVβ3 (551) - αVβ3(490)	3.11
7	2856.5971	EKSFTIKPVGFK-EKSFTIKPVGFK	αVβ3(417) - αVβ3(417)	2.17
8	2124.0699	LEVSVDSDQK-KEKETFK	αVβ3(605) - ProAgio (50)	842.21

Integrin αVβ3: ProAgio:: 1 : 0.7

$$ppm = 1.0 \times 10^6 \; \frac{(\text{measured mass} - \text{theoretical mass})}{\text{theoretical mass}}$$

Supplementary Table 2: Mass of the crosslinked peptides identified using pLink and corresponding regions in integrin αVβ3. Integrin αVβ3 and ProAgio were crosslinked at 1:0.7 ratio. δPPM is determined using the formula above mentioned.
